# Caspase-12 Silencing Attenuates Inhibitory Effects of Cigarette Smoke Extract on NOD1 Signaling and hBDs Expression in Human Oral Mucosal Epithelial Cells

**DOI:** 10.1371/journal.pone.0115053

**Published:** 2014-12-11

**Authors:** Xiang Wang, Ya-jie Qian, Qian Zhou, Pei Ye, Ning Duan, Xiao-feng Huang, Ya-nan Zhu, Jing-jing Li, Li-ping Hu, Wei-yun Zhang, Xiao-dong Han, Wen-mei Wang

**Affiliations:** 1 Department of Oral Medicine, Institute and Hospital of Stomatology, Nanjing University Medical School, Nanjing, China; 2 Department of Endodontics, Institute and Hospital of Stomatology, Nanjing University Medical School, Nanjing, China; 3 Department of Oral Pathology, Institute and Hospital of Stomatology, Nanjing University Medical School, Nanjing, China; 4 Department of Oral and Maxillofacial Surgery, Institute and Hospital of Stomatology, Nanjing University Medical School, Nanjing, China; 5 Immunology and Reproduction Biology Laboratory, Medical School, Nanjing University, Nanjing, China; 6 Jiangsu Key Laboratory of Molecular Medicine, Medical School, Nanjing University, Nanjing, China; 7 State Key Laboratory of Analytical Chemistry for Life Science, Nanjing University, Nanjing, China; H. Lee Moffitt Cancer Center & Research Institute, United States of America

## Abstract

Cigarette smoke exposure is associated with increased risk of various diseases. Epithelial cells-mediated innate immune responses to infectious pathogens are compromised by cigarette smoke. Although many studies have established that cigarette smoke exposure affects the expression of Toll-liked receptor (TLR), it remains unknown whether the nucleotide-binding oligomerization domain-containing protein 1 (NOD1) expression is affected by cigarette smoke exposure. In the study, we investigated effects of cigarette smoke extract (CSE) on NOD1 signaling in an immortalized human oral mucosal epithelial (Leuk-1) cell line. We first found that CSE inhibited NOD1 expression in a dose-dependent manner. Moreover, CSE modulated the expression of other crucial molecules in NOD1 signaling and human β defensin (hBD) 1, 2 and 3. We found that RNA interference-induced Caspase-12 silencing increased NOD1 and phospho-NF-κB (p-NF-κB) expression and down-regulated RIP2 expression. The inhibitory effects of CSE on NOD1 signaling can be attenuated partially through Caspase-12 silencing. Intriguingly, Caspase-12 silencing abrogated inhibitory effects of CSE on hBD1, 3 expression and augmented induced effect of CSE on hBD2 expression. Caspase-12 could play a vital role in the inhibitory effects of cigarette smoke on NOD1 signaling and hBDs expression in oral mucosal epithelial cells.

## Introduction

Cigarette smoke, including active smoking and passive smoking, has been implicated in many diseases, disability and death [1]. Cigarette smoke consists of more than 7300 chemical constituents, many of which are potent carcinogens and tumor promoters. A number of specific infections have been associated closely with cigarette smoke, including community-acquired pneumonia, tuberculosis, Helicobacter pylori infections, inflammatory bowel disease, invasive fungal infections, periodontitis, and oral candidiasis. Even though cigarette smoke directly mediates upregulation of bacterial virulence, the pro-infective effects of cigarette smoke are believed to result primarily from interference with host defense [2].

Innate immunity constitutes the first line of defense against microbe infection. As two main classes of innate immune receptors, the Toll-like receptors (TLR) and NOD-like receptors (NLR) serve as pattern recognition receptors that recognize conserved structures of pathogens, toxic compounds, or cellular damage known as “danger signals.” Depending on the adapter Receptor-interacting protein 2 (RIP2), NOD induces NF-κB activation and nuclear translocation. NF-κB activation promotes the production of proinflammatory cytokines, chemokines, and antimicrobial peptides. The human defensins, one group of small cationic antimicrobial peptides include the α-defensins of intestinal and neutrophil origin, and the β-defensins of skin, oral mucosa and other epithelia [3]. The human β defensins (hBDs) play important roles in innate immune and adaptive immune, such as antimicrobial activity, antitumor effect, chemoattractive effect and immunomodulation [4]. hBD1, 2, and 3 represent the main group of human defensins expressed and secreted by oral mucosal epithelial cells and have been most investigated.

So far the best characterized proteins of NLR members are nucleotide-binding oligomerization domain-containing protein 1 (NOD1) and NOD2. As one of intracellular pattern recognition receptors (PRRs), NOD1 plays a pivotal role in pathogen microbe clearance and tissue homeostasis of oral cavity, gastrointestinal, and respiratory tract. Sugawara et al. indicated that NOD1 and NOD2 in oral epithelial cells were functional receptors that induced antibacterial responses [5], [6].

Cigarette smoke directly activates epithelial cells and induces chemokine and inflammatory mediator release. Nevertheless, epithelia-mediated innate immune responses to infectious pathogens are compromised by cigarette smoke [7]. Although many studies have established that cigarette smoke exposure affects the expression of TLRs, study data about the effects of cigarette smoke exposure on NLRs remain scarce [8], [9], [10], [11], [12], [13]. Aldhous et al. have determined that Cigarette Smoke Extract (CSE) delays NOD2 expression and affects NOD2/RIP2 interactions in intestinal epithelial cells [14]. However, it remains unknown whether NOD1 expression is affected by cigarette smoke exposure.

Caspases are cysteinyl aspartate-specific proteases that play a pivotal role not only in the induction of apoptotic cell death but also in the inflammatory responses against microbial infection. Caspases are divided into three functional groups: apoptosis induction (Caspase-2, -3, -6, -7, -8, -9, and -10), inflammatory responses (Caspase-1, -4, -5, -11, and -12) and differentiation (Caspase-14). Caspase-1 is activated in the inflammasome, an intracellular protein complex that is formed by the recognition of intracellular ligands or cellular stresses by sensor molecules such as NOD-like receptors. Caspase-1 activation can induce the production of mature IL-1β/IL-18 and trigger pyroptosis. Under certain conditions, Caspase-11 is required for the activation of the caspase-1 inflammasome, referred to as the noncanonical inflammasome. In addition, Caspase-8 also contributes to the production of inflammatory cytokines [15]. Specially, only Caspase-12 can dampen the responses to bacterial infection and inhibit IL-1β, IL-18, and IFN-γ production. It had been confirmed that Caspase-12-deficiency not only enhanced bacterial clearance and sepsis resistance, but also augmented the production of antimicrobial peptides, cytokines, and chemokines to some pathogens [16], [17]. Previous studies have determined that cigarette smoke exposure or some components in cigarette smoke could up-regulate the expression of Caspase-12 [18], [19], [20], [21], while Caspase-12 could negatively modulate NOD1 signaling [17]. Based on these established evidences, we hypothesized that cigarette smoke may also have direct effect on NOD1 signaling and the production of antimicrobial peptides of human oral mucosal epithelial cells by up-regulating the expression of Caspase-12. The first goal of this study was thus to investigate whether CSE affected the expression of crucial molecules in NOD1 signaling pathway, including NOD1, RIP2, NF-κB and hBD1, 2, 3 in human oral mucosa epithelial cells. Our second focus was to verify the potentially inhibitory effect of Caspase-12 on NOD1 signaling and hBD1, 2, 3 in these cells following CSE exposure.

## Materials and Methods

### Reagents

Keratinocyte Serum-Free Medium (K-SFM) for Culture of Human Keratinocytes (Keratinocyte-SFM) was purchased from GIBCO (Invitrogen, Carlsbad, CA, USA), phosphatase inhibitor cocktail purchased from Roche (Mannheim, Germany), protease inhibitor cocktail was purchased from Fermentas UAB (Vilnius, Lithuania) and protein assay reagent and an enhanced chemiluminescent (ECL) kit were purchased from Pierce (Rockford, IL, USA). The following antibodies were used: rabbit anti-NOD1 antibody, rabbit anti-Caspase-12 antibody, mouse anti-RIP2 antibody, rabbit anti-p-NF-κB (p-p65) antibody, rabbit anti-NF-κB (p65) antibody, mouse anti-hBD1 antibody, and rabbit anti- hBD2 antibody were purchased from Abcam (Cambridge, UK); rabbit anti-hBD3 antibody was purchased from Novus (Littleton, CO, USA); rabbit anti-GAPDH antibody was purchased from Cell Signaling (Danvers, MA, USA). Dylight Fluor 488-labeled goat-anti-mouse secondary antibody and Alexa Fluor 555-labeled goat-anti-rabbit secondary antibody were purchased from Jackson ImmunoResearch (West Grove, PA, USA). Caspase-12 small interfering RNA (siRNA) and scrambled siRNA were purchased from GenePharma (Shanghai, China). Lipofectamine 2000 and Opti-MEM I medium was purchased from Invitrogen (Carlsbad, CA, USA). RevertAid First Strand cDNA synthesis kit was purchased from Fermentas UAB (Vilnius, Lithuania), and SYBR Green PCR Master Mix was purchased from Roche (Mannheim, Germany). The hBD1, 2, 3 ELISA Kits were purchased from Jingtian (Shanghai, China).

### Preparation of aqueous cigarette smoke extracts (CSE)

Aqueous CSE was prepared as previously described [22], [23]. Kentucky 3R4F research-reference filtered cigarettes (The Tobacco Research Institute, University of Kentucky, Lexington, KY), one of which contains 0.73 mg of nicotine and 9.4 mg of tar, were used for CSE preparation. A cigarette was smoked continuously by a peristaltic pump. Four cigarettes were bubbled through 40 ml of cell growth medium, and this solution, regarded as 100% strength CSE. The generated CSE solution was filtered (0.22 mm) to remove bacteria and large particles which was adjusted to a pH of 7.45 and used within 15 min after preparation. The content of nicotine in CSE was analyzed in the institutional laboratory using liquid chromatography-tandem mass spectrometry as previously described [24], [25], [26]. The 100% CSE contained 239±45 µg/ml of nicotine in three separate samples. Working dilutions of CSE (in the range of 0.5% to 8%) were made with culture medium expressed as a percentage (v/v %).

### Cell culture

Immortalized human oral mucosal epithelial (Leuk-1) cell line was a generous gift from Professor Li Mao at Department of Oncology and Diagnostic Sciences, University of Maryland Dental School, Baltimore, MD. The Leuk1 cell line was established from a dysplastic leukoplakia lesion adjacent to a squamous cell carcinoma of the tongue. It exhibits an immortalized but nontumorigenic phenotype [27]. The cell line was expanded and passaged in keratinocyte serum free medium [28]. This medium was supplemented with BPE (25 µg/ml), epidermal growth factor (0.2 ng/ml), CaCl_2_ (0.4 mM). The passaged cells were cultured in 37°C humidified air incubators with 5% CO_2_ which were stimulated with CSE of different concentration (0.5%, 1%, 2%, 4%, and 8%) in certain experiments.

### Western immunoblot analysis

Western blotting was performed as described [29]. Leuk-1 cells were washed twice with PBS and harvested by trypsinization. Cells were lysed in ice-cold lysis buffer containing 1% Nonidet P-40, 0.5% deoxycholate, 0.1% SDS, protease inhibitor cocktail, and phosphatase inhibitor cocktail. The lysates were incubated on ice for 30 min and centrifuged at 14,000 g, for 10 min, at 4°C, to remove cell debris. Total cellular protein was collected and protein concentration was measured. Next, 10 to 50 µg of total cell protein was separated by 10% SDS-PAGE gels and transferred from gels to polyvinylidene difluoride membranes (Millipore, Bedford, MA) by wet electroblotting. Membranes were blocked for 1 h at room temperature with 5% bovine serum albumin (BSA) in PBS-0.1% Tween 20 (PBST). After washing with PBST for three times, membranes were incubated with the primary antibodies (NOD1, Caspase-12, RIP2, p-NF-κB, and GAPDH antibodies were diluted 1:1,000 with PBST containing 5% BSA) overnight at 4°C. The next day, membranes were washed with PBST followed by 1 h incubation at room temperature with horseradish peroxidase-conjugated secondary antibodies (5,000-fold diluted with PBST containing 5% BSA). Following washing with PBST, immunostained protein bands were detected by using an enhanced chemiluminescence (ECL) assay kit and were visualized on FluorChem FC2 system (Cell Biosciences, Santa Clara, CA). Densitometric analyses of bands were performed using Image J software and the data of the target protein were normalized to those of the corresponding GAPDH (http://rsb.info.nih.gov/ij/). Data were normalized to GAPDH and expressed as the percentage or fold change compared with the corresponding control, which was set to 1 or 100.

### RNA extraction and real time quantitative reverse transcription PCR (qRT-PCR)

qRT-PCR assay was performed as described previously [17]. Total RNA was extracted from Leuk-1 cells by using TRIzol reagent (Invitrogen) according to the manufacturer's instructions, and 2μg of RNA was used to synthesize first-strand cDNA synthesis in 20 μl of reaction volume using RevertAid First Strand cDNA Synthesis Kit (Roche) according to the manufacturer's protocol. The primers used for the PCR amplifications are listed as follows: hBD1 forward TCA TTA CAA TTG CGT CAG CAG, reverse TTG CAG CAC TTG GCC TTC [30]; hBD2 forward TCC TCT TCT CGT TCC TCT TCA, reverse AGG GCA AAA GAC TGG ATG AC [30]; hBD3 forward CCA TTA TCT TCT GTT TGC TTT GCT C, reverse CCG CCT CTG ACT CTG CAA TAA TA [31]; Caspase-12 forward AAT GGA ATC TGT GGG ACC AA, reverse GAA CCA AAC AAT CCC AGC AC [32]; GAPDH forward TCA AGA AGG TGG TGA AGC AG, reverse CCC TGT TGC TGT AGC CAA AT [30]. Real-time PCR analyses ware performed using an ABI 7300 Real Time PCR System (Applied Biosystem, Foster City, CA), and PCR amplifications were performed using the SYBR Green PCR Master Mix (Roche) according to the manufacturer's instructions. Amplification conditions were as follows: 50°C for 2 min, 95°C for 10 min, 40 cycles of 95°C for 15 s, 58°C for 30 s, and 72°C for 30 s, followed by melting curve analysis, by which the specificity of primers was confirmed. The experiment was repeated three times. The data are expressed as relative mRNA levels and were normalized to GAPDH. Fold changes in expression of each gene were calculated by a comparative threshold cycle (Ct) method using the formula 2^−(ΔΔCt)^.

### Immunofluorescence, confocal microscopy and densitometry image analysis

Immunostaining was performed as described previously [33]. Briefly, Leuk-1 cells were collected and pipetted onto coverslips, which had been put in six-well culture plate in advance. After overnight incubation, Leuk-1 cells were washed with PBS and fixed in 4% paraformaldehyde for 15 min at room temperature. After being washed in PBS, the cells were permeabilized in 0.5% (v/v) Triton X-100 in PBS, washed, and blocked with 5% BSA in PBS-0.1% Tween 20 for 1 h at 37°C. Next, the cells were exposed overnight at 4°C to primary antibodies. Primary antibodies against the following proteins were used: NOD1 (1:100), Caspase-12 (1:200), RIP2 (1:50), phospho- NF-êB p65 (1:100), NF-êB p65 (1:100), hBD1 (1:100), hBD2 (1:100), and hBD3 (1:100). The next day, coverslips were washed with PBS and then incubated with Dylight 488 (green) or Alexa Fluor 555 (red) - labeled goat-anti-mouse or goat-anti-rabbit secondary antibody for 1 h at room temperature. To stain the nuclei, 4′, 6-diamidino-2-phenylindole (DAPI, Sigma) was added for 5 min, and slides were examined by a confocal laser scanning microscope (FluoView FV10i, Olympus, Japan). Densitometry image analysis was performed as previously reported with some modifications [34]. Five randomly selected discontinuous fields per slice were evaluated. The densitometry analysis of immunofluorescence results was performed by one blinded investigator using the Image J software. Briefly, the software was used to achieve the “gray image” and measure the optical density of the selected pixels within the region of interest (ROI). The calibration procedure was finished before image analysis. The mean value of the optical densities of all selected pixels was Mean Optical Density (MOD), which represented the corresponding fluorescence intensity of immunofluorescence staining.

### Enzyme-linked immunosorbent assay (ELISA)

To detect the amount of hBD1, 2, 3 produced by Leuk-1 cells, ELISA kits were used to measure the levels of hBD1, 2, 3 in cell culture supernatant. hBD1, 2, 3 standard was used to construct standard curves. The experiments were performed according to the manufacturer's recommendations. Absorbances were read at 450 nm and 570 nm using a microplate reader, the absorbance at 570 nm was subtracted from the absorbance at 450 nm.

### Preparation of siRNA and cell transfection

Caspase-12 silencing was obtained through transfecting FAM fluorescence-labeled siRNA, which was used to determine the transfection efficiency. Cell transfection was carried out by using siRNA as previously described [17], [35]. For Caspase-12 siRNA transfection, cells grew in 60 mm-diameter dishes and transfection was performed when cells were 50%∼70% confluent. For each dish, 100 pmol of Caspase-12 siRNA and 5 µl of Lipofectamine 2000 were diluted in 250 µl of Opti-MEM I reduced serum medium. Caspase-12 siRNA and Lipofectamine 2000 dilutions were then mixed and incubated at room temperature for 20 min before being added to each well containing cells and K-SFM (final concentration of Caspase-12 siRNA was 40 nM). The cells were incubated at 37°C in a CO_2_ incubator for 24 h, transfection medium was replaced by complete K-SFM, and the incubation continued for an additional 24 h before the addition of CSE. Controls were treated with scrambled siRNA and Lipofectamine 2000. The sequences of siRNAs are as follows [36]: Caspase-12 siRNA-1 sense 5′- GCA GUU AUA CAC GAG AUC ATT -3′ and antisense 5′- UGA UCU CGU GUA UAA CUG CTT -3′, Caspase-12 siRNA-2 sense 5′- GGC UCU UGC AAG GUA ACA UTT -3′ and antisense 5′- AUG UUA CCU UGC AAG AGC CTT -3′, scrambled siRNA sense 5′- UUC UCC GAA CGU GUC ACG UTT -3′ and antisense 5′- ACG UGA CAC GUU CGG AGA ATT -3′.

### Antimicrobial assay of culture supernatants of Caspase-12-silenced cells and control cells following CSE treatment

The antimicrobial assay of culture supernatants was performed as described previously [37]. *Candida albicans* strain ATCC 10231 was purchased from China Center of Industrial Culture Collection (CICC). *C. albicans* was incubated in yeast extract-peptone-dextrose (YPD) liquid media at 37°C overnight. Following various concentration of CSE treatment for 24 h, each of the culture supernatants of Caspase-12-silenced cells or control cells was then harvested, centrifuged and filtered. One hundred microliters each of the culture supernatants was added to 100 ml each of the *C. albicans* suspensions and incubated under 5% CO_2_ at 37°C for 1 h. After serial dilutions (until 10^4^-fold), each *C. albicans* suspension was then applied to YPD agar plates, incubated at 37°C for 24 h and subjected to colony counting. The antimicrobial activity of each culture supernatant was measured three times.

### Statistical analyses

Statistical analyses were performed using SPSS 15.0 (Chicago, IL). Values are expressed as the mean ± SE. Differences between the groups were analyzed via an unpaired *t*-test, and ANOVA was used to compare the differences between the different concentrations within the same treatment group. Two-tailed probability values of <0.05 were considered statistically significant. Error bars on images represent SE.

## Results

### CSE altered NOD1, Caspase-12, RIP2, and p-NF-κB expression in Leuk-1 cells

**Figure 1:**
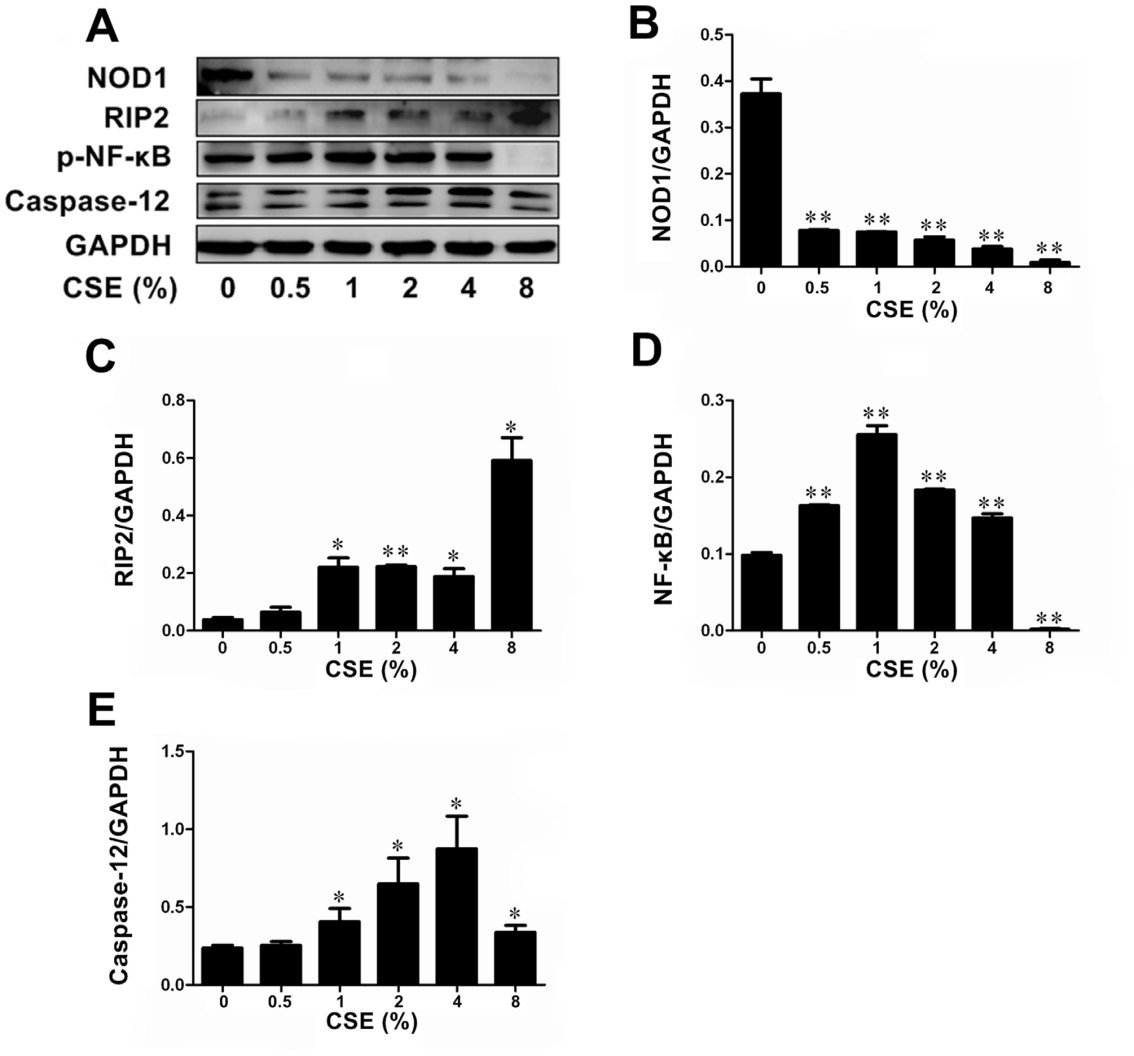
CSE exposure altered levels of NOD1, Caspase12, RIP2, and p-NF-κB protein expression in Leuk-1 cells. **A** Immunoblot bands indicated effects of CSE exposure on protein expression of NOD1, RIP2, p-NF-κB and Caspase12. **B** CSE treatment decreased the expression of NOD1 in a dose-dependent manner. **C** CSE treatment increased the expression of RIP2 in a dose-dependent manner. **D** p-NF-κB expression was activated by 0.5%∼4% CSE exposure, while 8% CSE treatment reduced p-NF-κB levels significantly. The p-NF-κB expression reached the highest level in Leuk-1 cells following 1% CSE exposure. **E** Caspase12 expression was activated by 1%∼8% CSE. Immunoblot band density data were expressed as means±SE (n = 3). Statistical significance: *P<0.05, **P<0.01 vs. cells without CSE treatment.

The first goal of this study was to investigate whether CSE affected the expression of crucial molecules of NOD1 signaling pathway in human oral mucosa epithelial cells. Leuk-1 cells were treated with various concentrations of CSE for 24 h. As shown by Western blotting results, the protein levels of NOD1 decreased with increasing concentration of CSE, while the protein levels of RIP2 elevated with increasing concentration of CSE (Fig. 1A, 1B and 1C). Relatively low concentrations of CSE treatment resulted in elevated p-NF-κB levels when comparing with the control group. The protein expression of p-NF-κB reached the highest level in Leuk-1 cells following 1% CSE treatment. However, p-NF-κB levels significantly decreased when exposed to higher concentrations of CSE (Fig. 1A and 1D). Caspase-12 expression was significantly increased in Leuk-1 cells following 1%∼8% CSE treatment, respectively (Fig. 1A and 1E).

**Figure 2:**
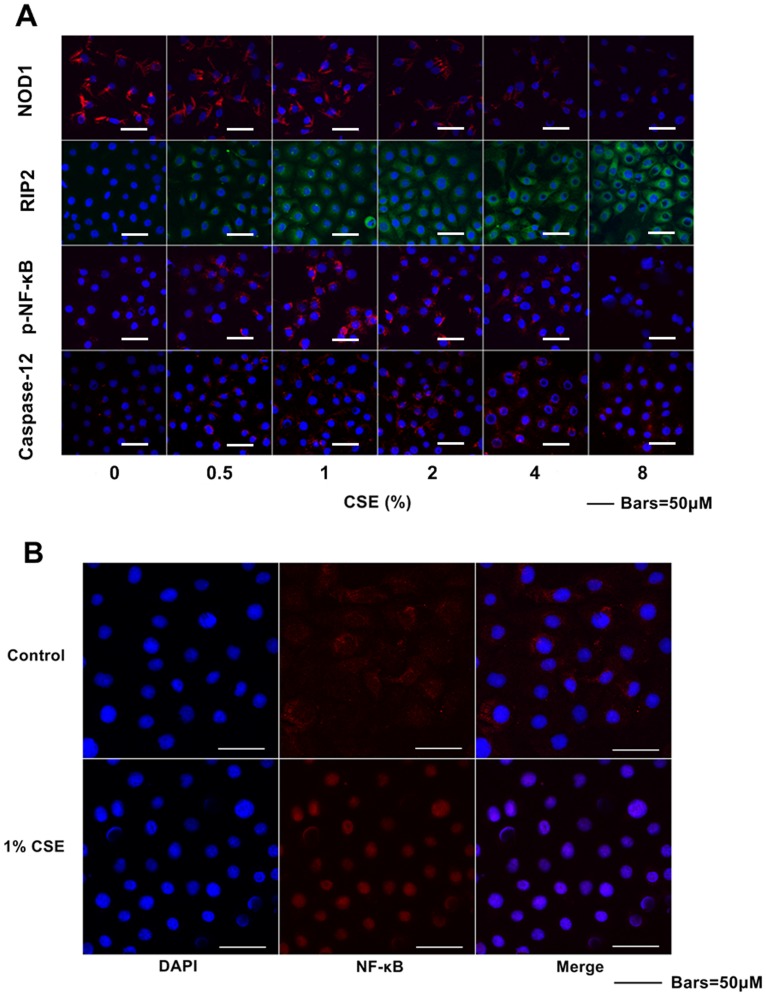
CSE exposure affected NOD1, Caspase12, RIP2, and p-NF-κB protein expression in Leuk-1 cells. Immunofluorescence staining was observed by confocal microscopy. **A** CSE exposure decreased the expression of NOD1 and increased the levels of RIP2. The expression of p-NF-κB was activated by relatively low concentrations of CSE, while 8% CSE treatment reduced p-NF-κB level. The expression of Caspase12 in Leuk-1 cells was activated by CSE treatment. **B** Compared with control cells, 1% CSE treatment induced marked translocation of NF-κB p65 subunit into nuclei.

Consistent with Western blotting results, immunofluorescence assays revealed that CSE down-regulated NOD1 protein levels and up-regulated RIP2 protein levels both in a dose-dependent manner. Relatively low concentrations of CSE elevated p-NF-κB levels, while relatively high concentrations of CSE reduced p-NF-κB levels. The p-NF-κB protein level reached the peak level following 1% CSE treatment. CSE activated Caspase-12 expression in Leuk-1 cells (Fig. 2A). As shown by confocal microscopy result, marked nuclear translocation of NF-κB p65 subunit was observed in Leuk-1 cells after 24 h following 1% CSE exposure (Fig. 2B).

### CSE regulated the expression and release of hBD1, 2, 3 by Leuk-1 cells

**Figure 3:**
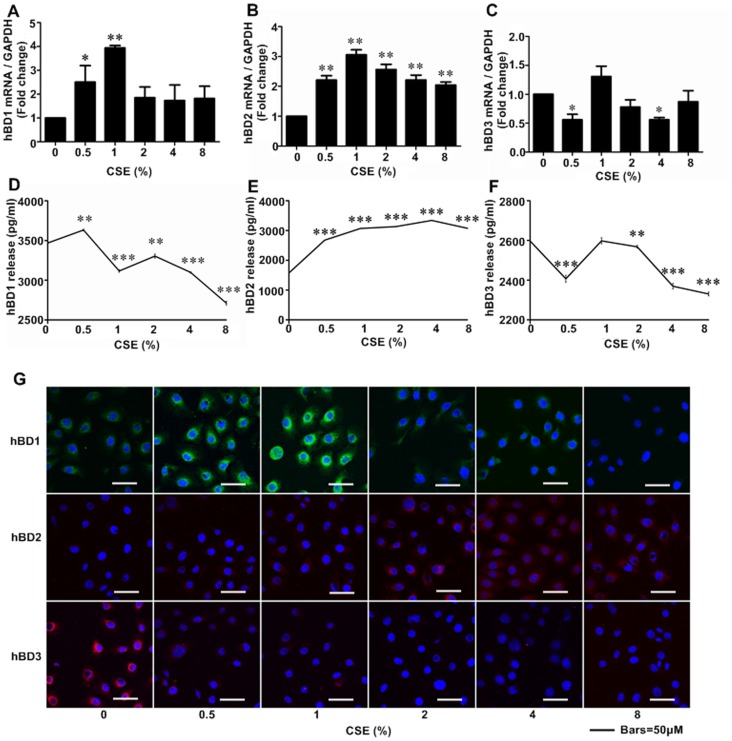
CSE regulated expression and release levels of hBD1, 2, 3 in Leuk-1 cells. **A** The real-time PCR results showed that hBD1 mRNA level significantly increased following 0.5% and 1% CSE treatment, especially for 1% CSE. **B** hBD2 mRNA levels were greatly up-regulated by CSE stimulation. **C** hBD3 mRNA levels were clearly decreased by 0.5% and 4% CSE. Following 1% CSE treatment, hBD2 and hBD3 mRNA expression in Leuk-1 cells reached the highest level. **D** ELISA analyses revealed that 0.5% CSE significantly increased hBD1 release and 1%∼8% CSE greatly decreased hBD1 release. **E** 0.5%∼8% CSE treatment significantly induced hBD2 release. **F** hBD3 release was significantly down-regulated by 0.5%, 2%, 4%, and 8% CSE treatment. **G** Immunofluorescence assay and confocal microscopy results showed that hBD1 protein level was markedly increased following 0.5% and 1% CSE treatment. Notably, hBD1 protein expression reached the highest level following 1% CSE exposure. CSE treatment greatly increased hBD2 protein expression and decreased hBD3 protein expression. The mRNA and ELISA data were expressed as means±SE (n = 3). Statistical significance: *P<0.05, **P<0.01, ***P<0.001 vs. cells without CSE treatment.

To clarify the effects of CSE on hBDs expression in human oral epithelial cells, qRT-PCR and immunofluorescence were performed to detect hBDs expression at mRNA and protein levels respectively. As shown by qRT-PCR results, relatively low concentrations of CSE treatment resulted in elevated hBD1 levels when comparing with control group. Interestingly, hBD1 mRNA level was up to the highest following the treatment of 1% CSE. However, hBD1 mRNA level significantly decreased when exposed to relatively higher concentrations of CSE (Fig. 3A). The real time qPCR results revealed that hBD2 mRNA levels up-regulated following CSE exposure (Fig. 3B). As shown by qRT-PCR results, 0.5% and 4% CSE treatment significantly down-regulated hBD3 mRNA level (Fig. 3C).

To further study the effects of CSE on hBDs releases from human oral epithelial cells, ELISA assays were performed to detect hBDs levels in the supernatant of Leuk-1 cells culture following CSE exposure. As Fig. 3D showed, 0.5% CSE treatment up-regulated hBD1 release, while CSE treatment of higher concentrations down-regulated hBD1 releases. On the contrary, CSE increased hBD2 releases in a dose-dependent manner (Fig. 3E). The hBD3 releases down-regulated following CSE treatment of 0.5%, 2%, 4% and 8% concentrations (Fig. 3F).

Consistent with qRT-PCR results, immunofluorescence assays indicated that hBD1 expression reached the highest level following the treatment of 1% CSE and down-regulated following relatively higher concentrations of CSE treatment. The Immunofluorescence assays revealed that hBD2 mRNA and protein levels up-regulated following CSE exposure. However, immunofluorescence staining results revealed CSE treatment completely down-regulated hBD3 protein level (Fig. 3G).

### The transfection of Caspase-12 siRNA caused Caspase-12 silencing at mRNA and protein levels

**Figure 4:**
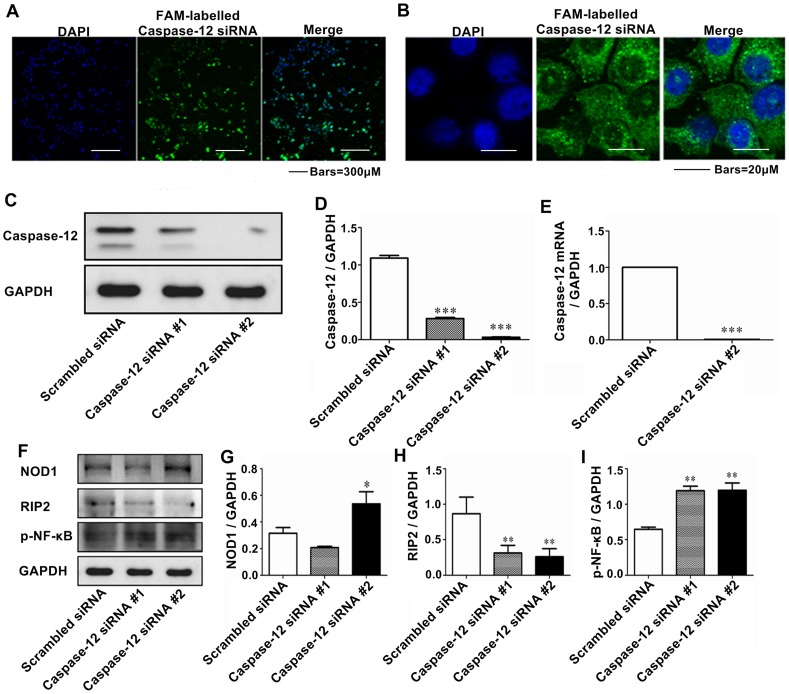
Caspase-12 silencing altered NOD1, RIP2 and p-NF-κB protein expression in Leuk-1 cells. **A** Immunofluorescence and confocal microscopy results showed that the transfection efficiency of Caspase-12 siRNAs was ∼95%. **B** FAM-labelled siRNAs indicated subcellular localization of cytoplasm, especially perinuclear area. **C** Western blot assay indicated protein level of Caspase-12 greatly decreased in Leuk-1 cells following RNA interference. **D** According to the image analysis result of immunoblot bands, compared with the NC group, the normalized protein level of Caspase-12 reduced remarkably in Caspase-12 siRNA-1 and -2 groups, especially for Caspase-12 siRNA-2. **E** Real-time PCR showed that the transfection of Caspase-12 siRNA-2 caused clear Caspase-12 silencing at mRNA level. **F** Western blot assay indicated Caspase-12 silencing altered NOD1, RIP2, and p-NF-κB protein expression. **G** Immunoblotting demonstrated that NOD1 expression significantly increased in Caspase-12-silenced cells compared with that in NC group. **H** Other than NOD1, Caspase-12 silencing remarkably reduced RIP2 level. **I** Alike to NOD1, p-NF-κB expression was up-regulated in Caspase-12-silenced cells. The immunoblot band density and mRNA data were expressed as means±SE (n = 3). Statistical significance: *P<0.05, **P<0.01, ***P<0.001 vs. scrambled siRNA-transfected cells.

To further examine the relationship between NOD1 signaling and Caspase-12, Caspase-12 was silenced by RNA interference. We selected two siRNA sequences from the region of Caspase-12 for evaluation. Non-silencing control siRNAs were synthesized using scrambled sequences as a negative control (NC) to assay the interference efficiency of Caspase-12 siRNA. These FAM labelled siRNAs were transfected into Leuk-1 cells. After 24 h, confocal microscopy showed that the transfection efficiency was ∼95% (Fig. 4A). FAM-labelled siRNAs indicated subcellular localization of cytoplasm, especially perinuclear area (Fig. 4B). As shown in Fig. 4C, in the Western blot assay, compared with the NC group, the normalized protein level of Caspase-12 reduced remarkably in Caspase-12 siRNA-1 and -2 groups. Especially Caspase-12 siRNA-2 had greater interference efficiency than caspase-12 siRNA-1. As shown in Fig. 4D, Caspase-12 levels in cells transfected with Caspase-12 siRNA-2 (40 nM) were ∼3% of that of NC group. Real-time PCR showed that the transfection of Caspase-12 siRNA-2 caused clear Caspase-12 silencing at mRNA level (Fig. 4E). Therefore Caspase-12 siRNA-2 was chosen in the following experiments.

### Caspase-12 silencing increased NOD1 and p-NF-κB expression and down-regulated RIP2 expression

RIP2 is a downstream component of Caspase-12 and NOD1 signaling is regulated by Caspase-12 in murine intestinal epithelial cells [17]. To determine whether NOD1 signaling was regulated by Caspase-12 in human oral epithelial cells, Leuk-1 cells were transfected with Caspase-12 siRNA-2 (40 nM). Immunoblotting demonstrated that NOD1 and p-NF-κB levels significantly increased in Caspase-12-silenced cells compared with that in NC group. Other than NOD1 and p-NF-κB, Caspase-12 silencing remarkably reduced RIP2 level (Fig. 4F, 4G, 4H and 4I).

### Caspase-12 silencing did not significantly alter the expression of hBD1, 2 and 3 at mRNA and protein levels

**Figure 5:**
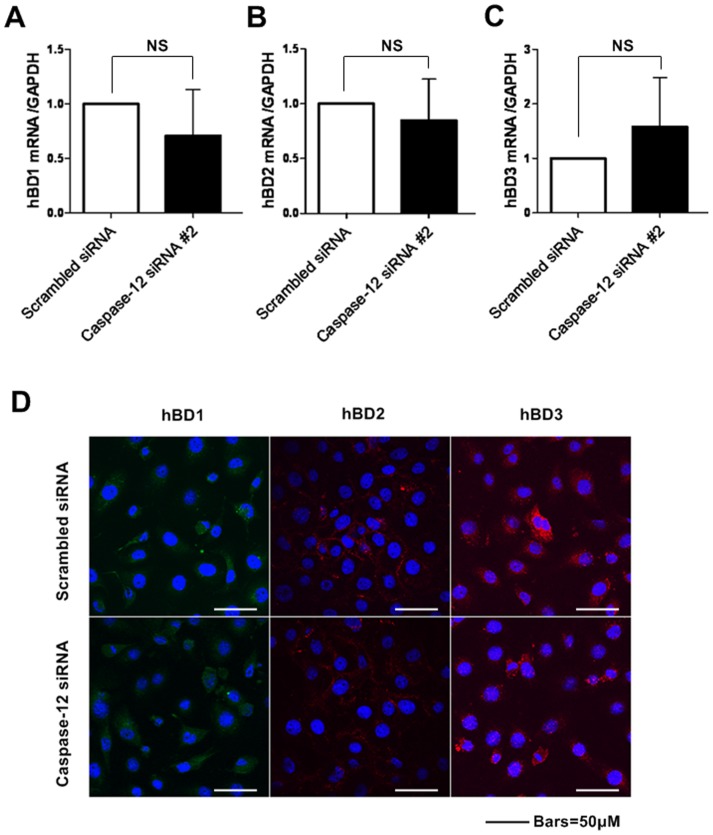
Caspase-12 silencing did not significantly alter hBD1, 2, 3 expression levels. **A-C** The real-time PCR results showed hBD1, 2, 3 mRNA levels in Caspase-12-silenced cells and that in control siRNA-transfected cells were comparable. **D** Immunofluorescence assay and confocal microscopy results showed that Caspase-12 silencing had no significant effect on hBD1, 2, 3 protein levels compared with the controls. n = 3. NS: no significant differences between groups.

Since Caspase-12 silencing increased NOD1 and p-NF-κB levels in Leuk-1 cells, we investigated whether Caspase-12 impacted hBD1, 2 and 3 at mRNA and protein levels. Firstly, we examined the relative alterations of hBD1, 2 and 3 at mRNA levels due to Caspase-12 silencing. As shown in Fig. 5A, 5B and 5C, no statistically significant change was observed in hBD1, 2 and 3 mRNA levels due to Caspase-12 silencing. We then analyzed whether Caspase-12 impacted hBD1, 2 and 3 at protein levels by immunofluorescence staining. Consistent with qRT-PCR results, Caspase-12 silencing also did not lead to significant change of hBD1, 2 and 3 protein expression compared with that in control cells (Fig. 5D).

### Caspase-12 silencing partially attenuated inhibitory effects of CSE on NOD1 and p-NF-κB expression

**Figure 6:**
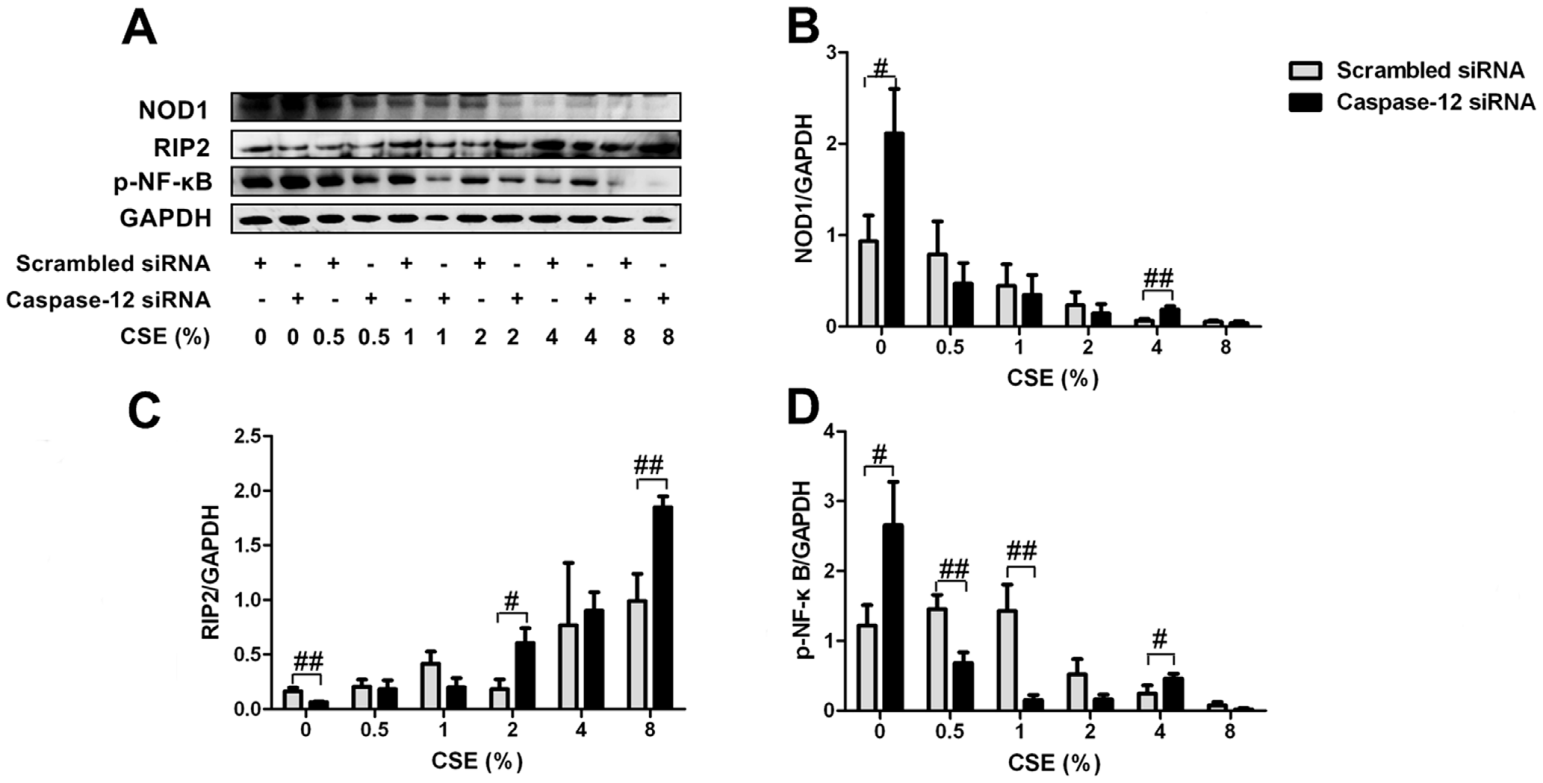
Caspase-12 silencing partially attenuated inhibitory effects of CSE on NOD1 and p-NF-κB expression. **A** Immunoblot bands indicated the protein expression of NOD1, RIP2, p-NF-κB in Caspase-12-silenced cells and controls exposed to CSE. **B** Immunoblot analysis showed that NOD1 protein level was significantly increased in Caspase-12-silenced cells compared with that in controls following 4% CSE treatment. **C** Caspase-12-silenced cells greatly increased RIP2 expression following 2% and 8% CSE treatment. **D** p-NF-κB level was greatly increased in Caspase-12-silenced cells compared with that in controls following 4% CSE treatment. Immunoblot band density data were expressed as means±SE (n = 3). Statistical significance: #*P*<0.05, ##*P*<0.01 vs. scrambled siRNA-transfected cells.

**Figure 7:**
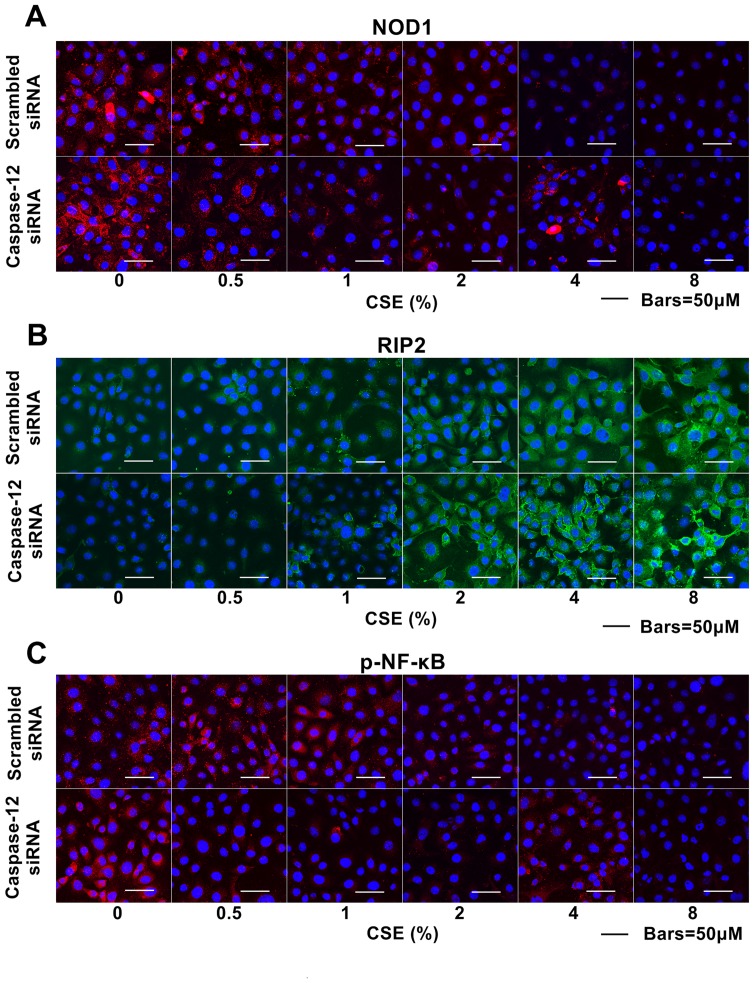
Caspase-12 silencing partially attenuated inhibitory effects of CSE on NOD1 and p-NF-κB expression. **A** Immunofluorescence assay and confocal microscopy results showed, compared with that in controls, NOD1 protein levels were remarkably increased in Caspase-12-silenced cells following 4% CSE treatment. **B** RIP2 levels were up-regulated in Caspase-12-silenced cells compared with that in controls following relatively high concentrations of CSE treatment. **C** p-NF-κB level greatly increased in Caspase-12-silenced cells compared with that in controls following 4% CSE treatment.

Caspase-12 has been reported to negatively regulate NOD1 signaling in enterocytes [17]. To confirm whether Caspase-12 inhibits NOD1 signaling following CSE exposure, we examined the impact of Caspase-12 silencing on NOD1 signaling pathway in Leuk-1 cells following CSE exposure. As immunoblotting analysis shown, NOD1 protein levels significantly increased in Caspase-12-silenced cells compared with that in controls following 4% CSE treatment (Fig. 6A and 6B). Caspase-12 silencing markedly increased RIP2 levels following 2% and 8% CSE treatment (Fig. 6A and 6C). Like NOD1, p-NF-κB level also greatly increased in Caspase-12-silenced cells compared with that in controls following 4% CSE treatment (Fig. 6A and 6D). These results confirmed that Caspase-12 silencing partially attenuated the inhibitory effect of CSE on NOD1 signaling pathway to a certain extent. Coincidentally, immunofluorescence results indicated that NOD1 protein levels significantly increased in Caspase-12-silenced cells compared with that in controls following 4% CSE treatment (Fig. 7A). Caspase-12 silencing significantly increased RIP2 levels following relatively high concentrations of CSE treatment (Fig. 7B). Immunofluorescence results indicated that p-NF-κB protein level significantly increased in Caspase-12-silenced cells compared with that in controls following 4% CSE treatment (Fig. 7C)

### Caspase-12 silencing abrogated the suppression of hBD1, 3 expression by CSE and augmented the induction of hBD2 expression by CSE

**Figure 8:**
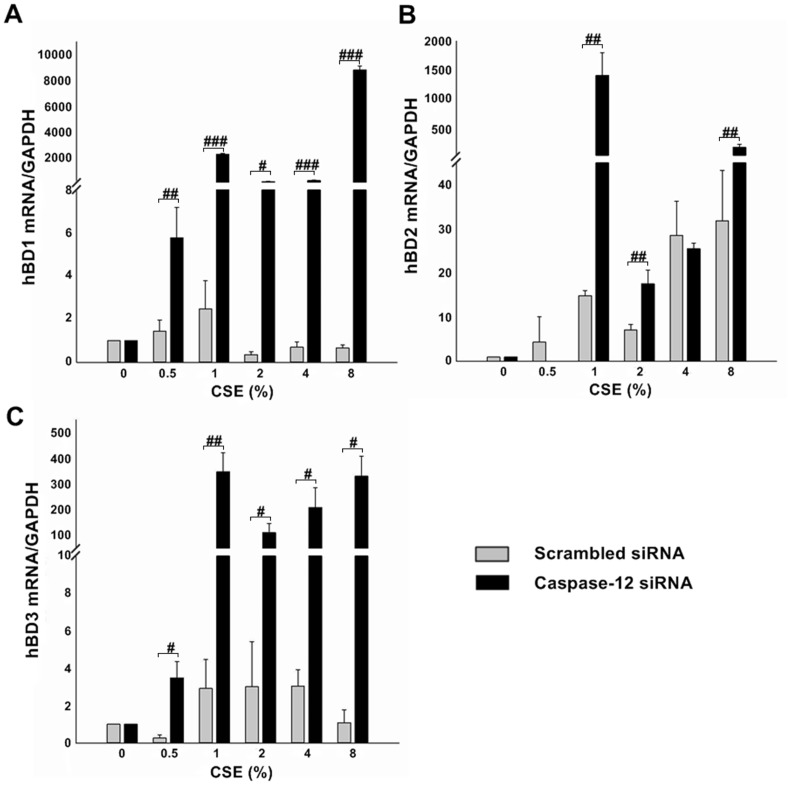
Caspase-12 silencing strikingly up-regulated hBD1, 2, 3 mRNA levels in Leuk-1 cells following CSE stimulation. **A** Real-time PCR results indicated that Caspase-12-silenced cells expressed 1,000-fold hBD1 mRNAs than the controls following 1% CSE treatment. Strikingly, Caspase-12-silenced cells expressed more than 8,000-fold hBD1 mRNAs than the controls following 8% CSE treatment. **B** The hBD2 mRNA levels increased and reached the highest level following 1% CSE treatment in Caspase-12-silenced cells, which expressed more than 1,000-fold hBD2 mRNAs than the controls. The hBD2 mRNA levels following 8% CSE treatment increased in Caspase-12-silenced cells, which expressed nearly 10-fold hBD2 mRNAs than the controls. **C** The hBD3 mRNA levels following 1% CSE treatment reached the peak level in Caspase-12-silenced cells, which expressed about 100-fold hBD3 mRNAs than the controls. Caspase-12-silenced cells following 8% CSE treatment also reached the peak level and expressed about 300-fold hBD3 mRNAs than the controls. The mRNA data were expressed as means±SE (n = 3). Statistical significance: #*P*<0.05, ##*P*<0.01, ###*P*<0.001 vs. scrambled siRNA-transfected cells.

In the next stage, we examined the impact of Caspase-12 silencing on CSE-stimulated hBD1, 2, 3 mRNA expressions in Leuk-1 cells. As shown in Fig. 8A, hBD1 mRNA levels increased and reached the highest level following 1% CSE treatment in control cells and then decreased following higher concentrations of CSE treatment. Caspase-12-silenced cells expressed 1,000-fold hBD1 mRNAs than the controls following 1% CSE treatment. Strikingly, the hBD1 mRNA levels following 8% CSE treatment reached the peak level in Caspase-12-silenced cells, which expressed more than 8,000-fold hBD1 mRNAs than the controls. As shown in Fig. 8B, hBD2 mRNA levels was up-regulated by CSE and reached the highest level following 8% CSE treatment in control cells. The hBD2 mRNA levels increased and reached the highest level following 1% CSE treatment in Caspase-12-silenced cells, which expressed more than 1,000-fold hBD2 mRNAs than the controls. The hBD2 mRNA levels following 8% CSE treatment increased in Caspase-12-silenced cells, which expressed nearly 10-fold hBD2 mRNAs than the controls. As shown in Fig. 8C, hBD3 mRNA levels decreased following 0.5% CSE treatment and increased following 4% CSE treatment and then decreased following 8% CSE treatment in control cells. The hBD3 mRNA levels following 1% CSE treatment reached the peak level in Caspase-12-silenced cells, which expressed about 100-fold hBD3 mRNAs than the controls. Caspase-12-silenced cells following 8% CSE treatment also reached the peak level and expressed about 300-fold hBD3 mRNAs than the controls.

**Figure 9:**
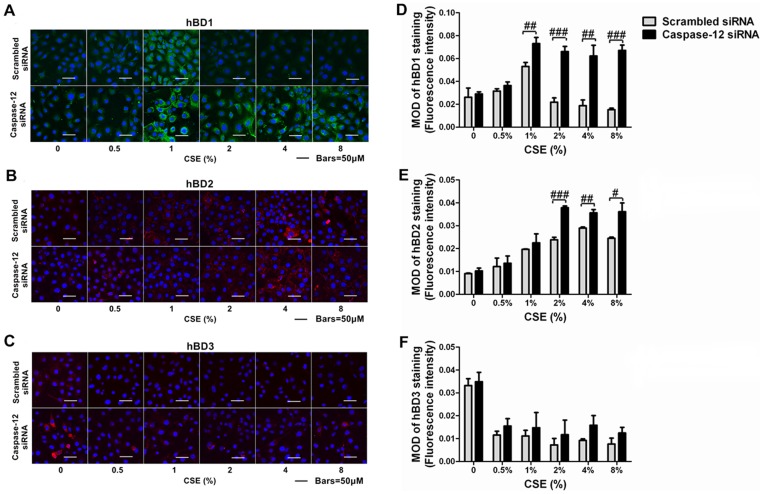
Caspase-12 silencing abrogated the suppression of hBD1, 3 protein expression by CSE and augmented the induction of hBD2 protein expression by CSE. **A–C** Immunofluorescence and confocal microscopy showed hBD1, 2, 3 staining in Caspase-12-silenced cells and control cells following various concentration of CSE treatment. **D** Densitometry results revealed that Caspase-12-silenced cells expressed markedly higher levels of hBD1 protein than the controls following 1%, 2%, 4% and 8% CSE treatment. **E** The hBD2 protein levels following 2%, 4%, and 8% CSE treatment were remarkably higher in Caspase-12-silenced cells than the control cells. **F** Following CSE exposure, Caspase-12-silenced cells expressed slightly higher levels hBD3 protein than the control cells. The MOD values of immunofluorescence staining were expressed as means±SE. Statistical significance: #*P*<0.05, ##*P*<0.01, ###*P*<0.001 vs. scrambled siRNA-transfected cells. MOD: mean optical density.

Since Caspase-12 silencing significantly increased hBD1, 2, 3 mRNA levels in Leuk-1 cells following CSE exposure, we investigated whether Caspase-12 silencing impacted hBD1, 2, 3 protein levels following CSE exposure by immunofluorescence staining (Fig. 9A, 9B and 9C). Consistent with qRT-PCR results, the image analysis of immunofluorescence results revealed that hBD1 protein expression increased and reached the highest level following 1% CSE treatment in control cells and then decreased following relatively higher concentrations of CSE treatment. To the contrary, Caspase-12-silenced cells expressed significantly higher levels of hBD1 than the controls following 1%, 2%, 4%, and 8% CSE treatment (Fig. 9D). As shown in Fig. 9E, CSE up-regulated hBD2 protein levels in control cells, while hBD2 protein levels following 2%, 4%, and 8% CSE treatment were remarkably higher in Caspase-12-silenced cells than the control cells. As shown in Fig. 9F, hBD3 protein levels in control cells decreased clearly following CSE exposure, while Caspase-12-silenced cells expressed slightly higher hBD3 levels than the controls following CSE exposure. However, the difference was not statistically significant.

### Caspase-12 silencing enhanced the antimicrobial activity of culture supernatants of CSE-exposed Leuk-1 cells

**Figure 10:**
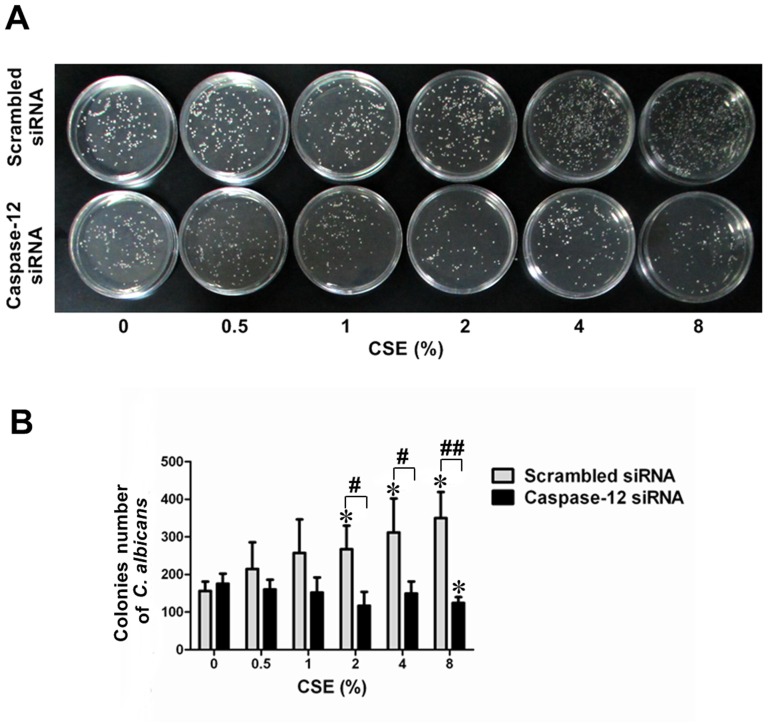
Caspase-12 silencing enhanced the inhibitory effect of culture supernatants of CSE-exposed Leuk-1 cells on *C. albicans*. Antimicrobial activities of culture supernatants of Caspase-12-silenced cells and control cells following CSE treatment were compared. **A** After the treatment with culture supernatants, *C. albicans* colonies were observed and counted at 24 h. **B** The colonies number of *C. albicans* clearly increased in NC group following the treatment with the culture supernatant of scrambled siRNA-transfected cells exposed to 2%, 4% and 8% CSE. The colonies number of *C. albicans* following the treatment with the culture supernatant of Caspase-12-silenced cells exposed to 2%, 4% and 8% CSE was remarkably fewer than that in NC group. The colonies number of *C. albicans* was expressed as means±SE (n = 3). Statistical significance: *P<0.05 vs. culture supernatants of cells not exposed to CSE; #*P*<0.05, ##*P*<0.01 vs. culture supernatants of scrambled siRNA-transfected cells.

As shown in Fig. 10, the culture supernatant of CSE-exposed control cells could not inhibit *C. albicans* colonies formation. The colonies number of *C. albicans* clearly increased in control groups following the treatment with the culture supernatant of control cells exposed to 2%, 4% and 8% CSE. Interestingly, the culture supernatant of CSE-exposed Caspase-12-silenced cells exhibited significantly higher antimicrobial activity to *C. albicans* than that of control cells. The colonies number of *C. albicans* following the treatment with the culture supernatant of Caspase-12-silenced cells exposed to 2%, 4% and 8% CSE was remarkably fewer than that in the corresponding NC group. These results indicated that Caspase-12 silencing enhanced the inhibitory effect of culture supernatants of CSE-exposed Leuk-1 cells on *C. albicans*.

## Discussion

Many studies have demonstrated that cigarette smoke alters the expression of PRRs, especially for TLRs [8]-[13]. However study data about the effects of cigarette smoke on NLRs remain very little. A recent study indicated that CSE delays NOD2 expression and affects NOD2/RIP2 interactions in intestinal epithelial cells [14]. As fundamental members of NLR family, NOD1 and NOD2 have very similar structures. Our data indicated for the first time that CSE could inhibit NOD1 signal pathway in oral mucosal epithelial cells.

There is a close relationship between cigarette smoke and many diseases. As is well known, smoking is one of the most important risk factors for periodontitis, second only to plaque [2]. A recent study indicated that NOD1 is critical for commensal-induced periodontitis [38]. Apart from periodontitis, cigarette smoke itself, or in combination with other factors, is a well recognized risk for oral candidiasis, oral leukoplakia and oral cancer [2], [39], [40], [41], [42].

In oral cavity, oral mucosal epithelium is the first tissue that encounters cigarette smoke. Very little study data existed about the effects of cigarette smoke on innate immune of oral mucosal epithelial cells. Given the vital role of NOD1 in innate immune and tissue homeostasis, the inhibitory effect of CSE on NOD1 expression could result in reduced antibacterial peptide production and oral diseases occurrence. NOD1 may be a potential therapeutic target for some diseases in future.

In the study, we found that CSE increased RIP2 expression in Leuk-1 cells. As an adapter, RIP2 acts a crucial role in NOD1-induced NF-κB activation. Moreover RIP2 also mediates cell apoptosis and autophagy according to previous studies [43], [44]. Recently, Wang et al. found that RIP1 expression remarkably increased in cigarette smoke-exposed mouse lung and significantly induced by CSE in human bronchial epithelial cells [45]. It is well known that receptor-interacting protein family consists of several members RIP1-4, which play a crucial role in cell survival signaling. Based on existing evidences, increased expression of RIP2 may result from CSE-induced cell damage.

Our early results indicated that low concentrations of CSE increased NF-κB expression in murine macrophages, while CSE of higher concentrations inhibited NF-κB activation [46]. Consistent with the study data, our present findings further confirmed CSE regulated-NF-κB activation or suppression is dependent on CSE concentrations. One of possible explanations is that the exposure to relatively low concentrations of CSE may lead to the cellular stress response to toxic compound stimulation. Organisms have developed an elaborate system of defensive molecules and survival signaling pathways to counteract various toxic and environmental stresses. If the adaptive response is unable to counteract adverse exposure, cells will be eliminated by death processes such as apoptosis [47].

The hBD is one of antimicrobial peptides that are expressed by the epithelia throughout the body including oral cavity. The expression of hBD1, 2, and 3 has been most investigated. These peptides are produced by oral epithelial cells and may control many commensal and pathogenic bacteria in oral cavity. hBD1 is constitutively expressed in epithelial cells and may be up-regulated by bacterial products. hBD2 is inductively expressed in epithelial cells and strongly up-regulated *in vitro* by commensal, pathogenic bacteria as well as proinflammatory cytokines. hBD3 is expressed in normal epithelium and is up-regulated by bacteria, IFN-γ and growth factors [48].

Study data on effects of cigarette smoke on hBD1 expression are little. Wolgin et al. found that the expression of hBD1 and hBD2 mRNA significantly reduced in gingival samples of smokers compared to that in non-smokers [30]. An early study indicated that mouse β defensin (mBD) 1 expression decreased in cigarette smoke-exposed mice, while the expression of mBD2 and mBD3 was greatly elevated in the lungs of cigarette smoke-exposed mice compared with air-exposed mice [49]. Some studies indicated that CSE or whole cigarette smoke exposure modulates hBD2 and hBD3 mRNA by human gingival epithelial cells *in vitro* [10], [11]. The current result indicated that CSE could modulate hBD1, 2, and 3 expression levels in Leuk-1 cells. hBD1 expression in Leuk-1 cells was activated by relatively low concentrations of CSE and suppressed by relatively high concentrations of CSE. Otherwise, our results indicated CSE significantly increased hBD2 expression and inhibited hBD3 levels in Leuk-1 cells. In the present results, the difference between hBDs mRNA expression levels in Leuk-1 cells and hBDs protein levels in the supernatant was observed. On the one hand, the difference could be explained by the regulation of antimicrobial peptide expression at transcriptional, post-transcriptional and post-translational levels [50], [51]. On the other hand, this difference may originate from the modulation of hBDs at secretory level by epithelial cells themselves, which released distinct amount of hBDs to control defensive responses to varying extents.

Many study results have confirmed that cigarette smoke or some components in cigarette smoke can activate Caspase-12 expression [18], [19], [20], [21]. In accordance to previous studies, our results suggested that CSE treatment could increase the expression of Caspase-12 in oral mucosal epithelial cells. Caspase12 is a crucial molecule associated with endoplasmic reticulum (ER) stress-induced apoptosis and inflammasome activation [52]. LeBlanc et al. found that Caspase-12 modulates negatively NOD1 signaling in mouse colonic epithelial cells. Mechanistically, Caspase-12 binds to RIP2 and displaces Traf6 from NOD1 signaling complex. As a result, its ubiquitin ligase activity is inhibited and NF-kB activation is blunted [17]. Supporting their findings, our results indicated that Caspase-12 silencing down-regulated RIP2 expression in Leuk-1 cells. Intriguingly, our results further showed that Caspase-12 silencing up-regulated the expression of NOD1 and NF-kB. These findings suggested clearly that Caspase-12 is a negative regulator of NOD1 signaling. Further studies are needed to investigate the complicated interacting mechanism between Caspase-12 and crucial molecules in NOD1 signal pathway.

The present study in Leuk-1 cells showed that Caspase-12 silencing partially attenuated inhibitory effects of CSE on NOD1 and p-NF-kB protein expression. After 4% CSE treatment, both NOD1 and p-NF-κB levels significantly increased in Caspase-12-silenced cells compared with that in controls. Therefore, these results indicated that Caspase-12 could be involved in the inhibitory effect of CSE on NOD1 signaling in oral mucosal epithelial cells.

**Figure 11:**
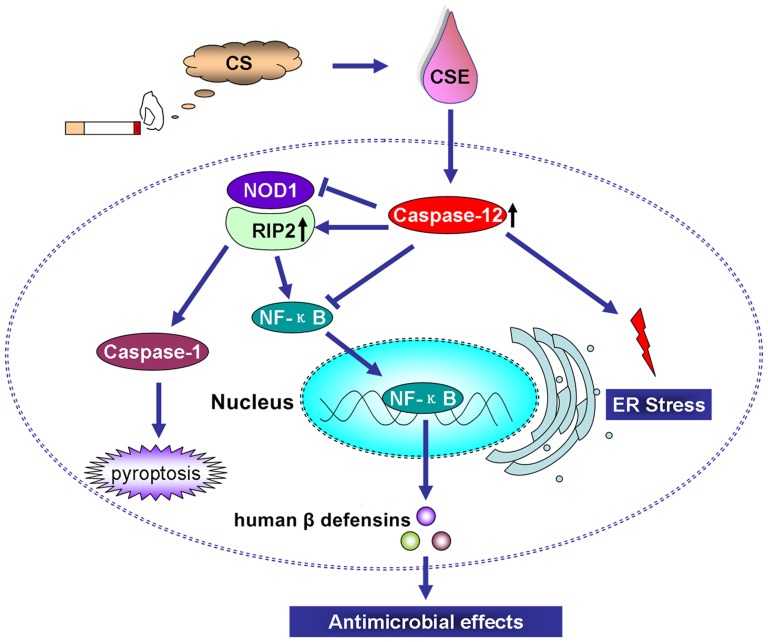
Schematic depiction of the potential mechanism by which CSE-induced Caspase-12 activation dampens the defense response of oral mucosal epithelial cells to pathogenic microorganism through inhibition of the NOD1 signaling and hBDs production. Mechanistically, CSE activated intracellular Caspase-12, which negatively regulated NOD1 signaling by suppressing NOD1 and NF-κB and inducing RIP2. As a part of downstream effectors of the signaling pathway, hBDs production was subsequently inhibited and the defense response to pathogens was dampened. CS: cigarette smoke; CSE: cigarette smoke extract.

In the present study we found for the first time that Caspase-12 silencing abrogated the suppression of hBD1, 3 expression levels by CSE and augmented the induction of hBD2 expression by CSE. Moreover our results indicated that Caspase-12 silencing enhanced the inhibitory effect of culture supernatants of CSE-exposed Leuk-1 cells on *C. albicans*, a common conditioned pathogen in oral cavity. LeBlanc et al. confirmed that Caspase-12-deficient enterocytes after infection hyper-produced antimicrobial peptides, specifically mBD1, a functional homolog of hBD1 [17]. LeBlanc and colleagues' results provide a clue that Caspase-12 regulates antimicrobial peptide production following infection stimulation. Coincidentally, our results suggested that Caspase-12 regulates antimicrobial peptide production following CSE stimulation. Antimicrobial peptide production may up-regulated in the absence of Caspase-12. Mechanistically, CSE activated intracellular Caspase-12, which negatively regulated NOD1 signaling by suppressing NOD1 and NF-κB expression and inducing RIP2 expression. As a part of downstream molecules of the signaling, hBDs production was subsequently inhibited and the defense response of human oral mucosal epithelial cells to pathogens was dampened (Fig. 11). Saleh et al. confirmed that Caspase-12-deficient mice enhanced bacterial clearance and sepsis resistance. Caspase-12 is detrimental to *in vivo* handling of systemic bacterial infections and predisposes to sepsis, thereby making it a potentially important target for future therapeutic strategies [53].

All together, CSE could suppress NOD1 signaling and modulate the downstream hBDs production in Leuk-1 cells. Caspase-12 silencing partially attenuated the inhibitory effects of CSE on NOD1 signaling and abrogated the suppression of CSE on hBDs expression. Caspase-12 silencing could enhance the antimicrobial activity of CSE-exposed cells. Caspase-12 may be a potential therapeutic target for some infectious and inflammatory diseases in future.

## Acknowledgments

We thank Professor Li Mao (School of dentistry, University of Maryland, USA) for his kind provision of oral mucosal epithelial (Leuk-1) cell line. We also thank Professor Wantao Chen (Department of Oral and Maxillofacial Surgery, Ninth People's Hospital, School of Stomatology, Shanghai Jiao Tong University School of Medicine, China) for his kind help to our experiment.
